# Interleukin 17 Receptor A Modulates Monocyte Subsets and Macrophage Generation In Vivo

**DOI:** 10.1371/journal.pone.0085461

**Published:** 2014-01-15

**Authors:** Shuwang Ge, Barbara Hertel, Nathan Susnik, Song Rong, Anna M. Dittrich, Roland Schmitt, Hermann Haller, Sibylle von Vietinghoff

**Affiliations:** 1 Department of Medicine, Hannover Medical School, Hannover, Germany; 2 Department of Pediatrics, Hannover Medical School, Hannover, Germany; 3 Department of Nephrology, Tongji Hospital, Huazhong University, Wuhan, China; University of Washington, United States of America

## Abstract

Interleukin (IL)-17A signaling via Interleukin 17 receptor A (*Il17ra*) contributes to the inflammatory host response by inducing recruitment of innate immune cells, but also plays a role in homeostatic neutrophilic granulocyte regulation. Monocytes, the other main innate immune cell, have a longer life span and can pursue multiple differentiation pathways towards tissue macrophages. Monocytes are divided into two subpopulations by expression of the Ly6C/Gr1 surface marker in mice. We here investigated the role of *Il17ra* in monocyte homeostasis and macrophage generation. In *Il17ra^-/-^* and in mixed bone marrow chimeric wt/*Il17ra^-/-^* mice, the concentrations of circulating *Il17ra^-/-^*Gr1^low^ monocytes were significantly decreased compared to wt cells. Pulmonary, splenic and resident peritoneal *Il17ra^-/-^* macrophages were significantly fewer than of wt origin. Bone marrow progenitor and monocyte numbers were equal, but the proportion of *Il17ra^-/-^*Gr1^low^ monocytes was already decreased at bone marrow level. After monocyte depletion, initial Gr1^high^ and Gr1^low^ monocyte regeneration of *Il17ra^-/-^* and wt cells was very similar. However, *Il17ra^-/-^*Gr1^low^ counts were not sustained. After labeling with either fluorescent beads or BrdU, *Il17ra^-/-^*Gr1^high^ monocyte transition to Gr1^low^ cells was not detectable unlike wt cells. Monocyte recruitment in acute peritonitis, which is known to be largely due to Gr1^high^ cell migration, was unaffected in an identical environment. Unilateral ureteral obstruction induces a less acute inflammatory and fibrotic kidney injury. Compared to wt cells in the same environment, *Il17ra^-/-^* macrophage accumulation in the kidney was decreased. In the absence of *Il17ra* on all myeloid cells, renal fibrosis was significantly attenuated. Our data show that *Il17ra* modulates Gr1^low^ monocyte counts and suggest defective Gr1^high^ to Gr1^low^ monocyte transition as an underlying mechanism. Lack of *Il17ra* altered homeostatic tissue macrophage formation and diminished renal inflammation and fibrosis. *Il17ra* appears to be a novel modulator of monocyte phenotype and possible therapeutic target in renal fibrosis.

## Introduction

Monocytes are innate immune cells that frequently mediate early immune response [1]–[3]. Life span is considerably longer than that of the other main innate myeloid cells, neutrophilic granulocytes, and monocytes can differentiate into macrophages with heterogeneous functions regarding cytokine production and antigen presentation in the target tissues. While some recruitment mechanisms of granulocytes and monocytes are similar, surface receptor expression [4] and mechanisms of mobilization from the bone marrow differ substantially [3].

Two CD115 (M-CSF-receptor)^+^ monocyte subpopulations can be distinguished in mice [1]–[3]. Surface molecules are differentially expressed, including the fractalkine receptor CX3CR1, the chemokine receptor CCR2, the GPI linked surface molecule Ly6C (also detected by the Gr1 antibody) and the integrin subunit CD11c [5], [6]. The two main subpopulations are Gr1^high^CX3CR1^low^CD11c^low^, sometimes termed “inflammatory” and Gr1^low^CX3CR1^high^CD11c^high^, sometimes termed “resident” monocytes [1]–[3]. Pharmacological blockade of the M-CSF receptor preferentially depletes Gr1^low^ monocytes [7], [8]. Recently, a role for the nuclear receptor NUR77 in maintenance of monocyte counts, most markedly the Gr1^low^ subtype, has been reported [9].

A number of observations suggest that most circulating Gr1^low^ cells develop from Gr1^high^ monocytes in the periphery [10]–[12]. While a mechanism driving this transition remains to be determined, the evidence includes that the proportion of Gr1^high^ cells is higher in bone marrow than peripheral blood, that Gr1^high^ cells reappear before Gr1^low^ cells after monocyte depletion [10], [11], and that Gr1^high^ cells labeled with fluorescent latex beads [13] or BrdU [12] can transition to Gr1^low^ monocytes.

Both monocyte subtypes contribute to tissue macrophages and myeloid antigen presenting cells. For generation of many macrophage types, especially in acute inflammation, Gr1^high^ monocytes appear to be the predominant subtype [1]–[3]. However, adoptive transfer experiments have demonstrated that Gr1^low^ monocytes give rise to splenic myeloid dendritic cells [14] and pulmonary macrophages [15] and very recently, with the use of novel genetic models, a significant contribution of Gr1^low^CX3CR1^high^ monocytes to resident macrophage populations in many organs has been documented [12]. Macrophages in the atherosclerotic aorta develop from both subtypes [16]. In acute inflammation in listerial peritonitis, Gr1^low^ monocytes are responsible for the immediate host response [17].

The T cell cytokine IL-17 participates in regulation of innate immunity. Neutrophil recruitment is enhanced by IL-17 e.g. in encephalitis, ischemia-reperfusion injury and solid allograft rejection [18], [19]. However, evidence of a direct IL-17 effect on neutrophils is lacking and current data rather suggest indirect action, e.g. via epithelial cells [18], [19]. Regarding monocytes and macrophages, we have previously demonstrated that IL-17 receptor A (*Il17ra*) that is required for both IL-17A and IL-17F [20], but also IL-17C and IL-17E signaling is expressed [21], [22]. Peritoneal macrophage accumulation and CD11c expression in acute peritonitis were decreased in IL-17 and IL-17 receptor A deficient (*Il17ra^-/-^*) mice [21]. Monocytes differentially express *Il17ra* and macrophage cytokine secretion is influenced by IL-17A [23].

We here investigated whether or not IL-17 signaling influences monocyte homeostasis, subpopulations and macrophage generation at rest and during inflammation *in vivo*. Our results indicate a significant impact of monocyte IL-17 receptor expression on monocyte counts and macrophage generation at an individual cell level.

## Materials and Methods

### Animals

Wild-type (wt, CD45.2) C57BL/6 and congenic B6.SJL-*Ptprc^a^Pepc^b^*/BoyJ (CD45.1) mice were from Jackson Labs (Bar Harbor, ME), mice lacking IL-17 receptor (*Il17ra^-/-^*) from Amgen (Thousand Oaks, CA) and genotyped by PCR. *Il17ra^-/-^* mice were >95% C57BL/6 background. Mice were kept in specific-pathogen-free conditions. Blood was collected via tail bleeds into EDTA-coated capillary tubes and leukocyte counts were measured by an automated analyzer (ScilVetABC, Viernheim, Germany). Animal experiments were approved by Landesamt für Verbraucherschutz und Lebensmittelsicherheit, Lower Saxony, Germany (33.9-42502-04-10/0253, 33.9-42502-04-08/1434). All surgery was performed under ketamine/xylazine anaesthesia and all efforts were made to minimize suffering.

### Bone marrow transplantation

Lethal irradiations were performed in a ^137^Cs irradiator (10Gy), and mice were reconstituted with un-fractioned bone marrow from wt (CD45.1) and *Il17ra^-/-^* (CD45.2) mice at a 1∶1 ratio verified by flow cytometry into wt (CD45.2) mice. Mice were treated with trimethoprim-sulfomethoxazole in drinking water for two weeks after transplantation. Experiments were conducted 6-12 weeks after bone marrow transplantation.

### Monocyte depletion by liposomal clodronate and labeling with latex microspheres

Liposomal clodronate was kindly provided by Dr. van Rooijen [24]. Mice were injected intravenously with 200 µl of clodronate or control PBS liposomes (5 mg/ml). This depleted monocytes both in peripheral blood and bone marrow (data not shown). FITC-labeled latex microspheres (0.5 µm, Polysciences, Eppelheim, Germany) were injected i.v. at a dose of 10 µl/mouse.

### Thioglycollate-induced peritonitis and unilateral ureteral obstruction

For induction of peritonitis, 1 ml of 3% thioglycollate (Sigma-Aldrich, St. Louis, MO) was injected i.p., and cells were recovered after 10 h or 3 days by washing twice with 5 ml PBS.

Unilateral ureteral obstruction surgery was performed and tissues analyzed on day 7 as described [25]. Anti-α-Smooth Muscle Actin-Alexa547 (1A4, Sigma, St. Louis, MO, USA). Images were obtained with a Zeiss Axioplan-2 microscope with 20× and 40× original magnification using AxioVision 4.6 (Zeiss, Jena, Germany). Quantification was conducted with NIH ImageJ. Values represent means of 3-6 high power fields/animal.

### Cell preparation and staining for flow cytometry

Single cells suspensions from blood, bone marrow, spleen, kidney and lung were prepared. Lungs and kidneys was digested with a cocktail of 125 U/ml collagenase XI, 450 U/ml collagenase I, 60 U/ml DNase I, and 60 U/ml hyaluronidase (Sigma-Aldrich) as described [21]. For assessment of TNFα production, monocytes from mixed bone marrow chimeric mice were stimulated for 60 min with 1 µg/ml LPS (E.coli, Sigma) at 37°C in EDTA-anti-coagulated whole blood.

The following antibodies were used: Anti-mouse lineage cocktail (BD Biosciences), CD3 (17A2), CD11b (M1/70), CD11c (N418), CD19 (6D5), CD34 (RAM34), CD45 (30-F11), CD45.1 (A20), CD45.2 (104), CD103 (2E7), CD115 (AFS98), CD117 (2B8), Gr1 (RB6-8C5), F4/80 (BM8), IL-17ra (PAJ-17R), Ly6C (HK1.4), Sca-1 (D7), TNFα (MP6-XT22). Antibodies were purchased from BD Biosciences (Heidelberg, Germany), BioLegend (San Diego, CA), eBioscience (San Diego, CA), or Invitrogen (Carlsbad, CA). Near infrared LIVE/DEAD® Fixable Dead Cell Stain Kit (Invitrogen, Carlsbad, CA), BD-Fix-Perm and BrdU flow kit (BD Pharmingen, San Jose, CA) were used according to the manufacturer's instructions. Flow cytometry analysis was performed on a BD Biosciences FACSCanto or LSRII, cells were sorted using a FACSAria. Data were analyzed using FlowJo software (Tree Star, Ashland, OR). Monocytes were defined as CD115^+^ leukocytes and subdivided by genotype with use of CD45.1 and CD45.2 markers before analysis of their phenotype (Gr1, Ly6C etc.) was performed.

### mRNA isolation and qPCR

Bone marrow CD11b^+^CD115^+^ monocytes were sorted (FACS-Aria) for Ly6C expression and mRNA was isolated using NucleoSpin® RNAII Kit (Macherey-Nagel, Duren, Germany) and reversely transcribed with M-MLV-RT (Promega, Mannheim, Germany) according to the manufacturer's instructions. Realtime PCR was performed on a LightCycler480 using Sybr-Green (Roche, Grenzach-Wyhlen, Germany). Primers were selected using PrimerBank as follows: Il17ra: fw: AGTGTTTCCTCTACCCAGCAC, rev: GAAAACCGCCACCGCTTAC Il17rb: fw: GGCTGCCTAAACCACGTAATG, rev: CCCGTTGAATGAGAATCGTGT, Il17rc: fw: GCTGCCTGATGGTGACAATGT, rev: TGGACGCAGGTACAGTAAGAAG, Il17re: fw: TCTGGCAGTCAATACGCTTCA, rev: GGTTTCGCAGGGTGTGAGT, collagen I: fw: TGT CCC AAC CCC CAA AGA C, rev: CCC TCG ACT CCT ACA TCT TCT GA, CTGF: fw: AAG ACC TGT GGA ATG GGC, rev: TGG TGC AGC CAG AAA GCT C, fibronectin: fw: GCA CAG GGG AAG AAA AGG AG, rev: TTG AGT GGA TGG GAG GAG AG, HPRT fw: CAGTCCCAGCGTCGTGATTA, rev: AGCAAGTCTTTCAGTCCTGTC. Products were confirmed by melting curve and gel electrophoresis. Transcript levels were normalized to HPRT using the ΔCt method.

### Statistical analysis

Two-tailed t-test or ANOVA (for more than two data points, as indicated in figure legends) with appropriate post-hoc test were used. Results from mixed chimeric mice were analyzed using paired t-test or repeated measures ANOVA if more than two measures were compared. Data are expressed as mean±SEM. P-values <0.05 were considered significant and are indicated in the figures as *p<0.05, **p<0.01, and ***p<0.001.

## Results

### Gr1^low^ monocyte deficiency in the absence of IL-17 receptor A

**Figure 1:**
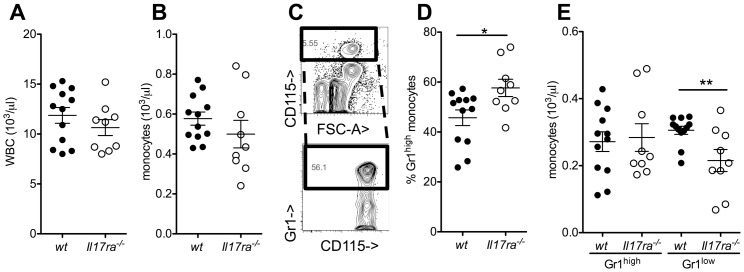
Decreased Gr1^low^ monocyte counts in IL-17 receptor A deficient mice. (**A,B**) Peripheral blood total white cell (A) and monocyte (B) counts were assessed by an automated analyzer in wild type (wt) and Interleukin-17 receptor A-deficient (*Il17ra^-/-^*) mice (n = 9–12). (**C–E**) Gr1^high^ and Gr1^low^ monocyte subgroups were analyzed by flow cytometry after gating for CD11b^+^CD115^+^ events (example in C, D: proportion of Gr1^high^ monocytes and E: absolute concentrations in wt and *Il17ra^-/-^* mice, n = 9–12, t-tests).

To test for a role of IL-17 receptor A in monocyte homeostasis, we analyzed peripheral blood monocytes in wildtype (wt) and *Il17ra^-/-^* mice. Total white blood counts and the concentration of monocytes were very similar (figure 1A, B), but the proportion of Gr1^high^ monocytes was significantly elevated in *Il17ra^-/-^* mice (figure 1C,D). This was not caused by an increase in Gr1^high^, but a decrease in Gr1^low^ absolute monocyte counts (figure 1E).

**Figure 2:**
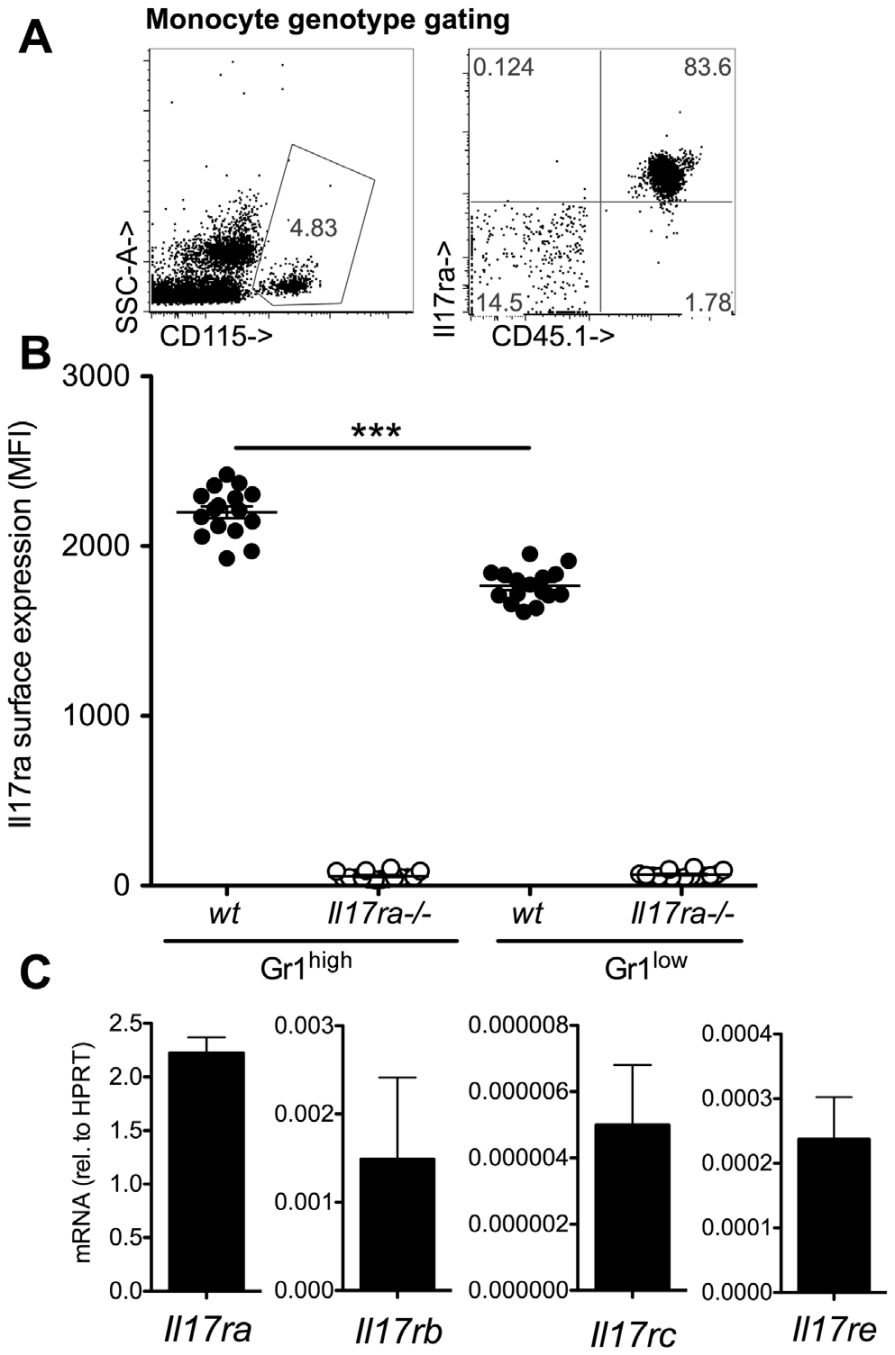
Monocyte characterization in mixed chimeric wt/IL-17 receptor A deficient mice. Monocyte analysis in mixed bone marrow chimeric wt(CD45.1)/*Il17ra^-/-^*(CD45.2) mice (recipients: CD45.2 wt mice). (**A**) Monocytes were gated by CD115 and IL-17 receptor expression was assessed as control of complete chimerism. (**B**) Gr1^high^ and Gr1^low^ monocytes in mixed chimeric mice were analyzed for the intensity of *Il17ra* expression (MFI, n = 16 from 3 independent transplantations, Bonferroni after One-way-ANOVA). (**C**) Gr1^high^ monocytes were sorted from bone-marrow and expression of IL-17 receptor subunits analyzed by qPCR (means of n = 2 independent cell sorts).

To dissect between systemic effects of IL-17 receptor deficiency and cell intrinsic effects in *Il17ra^-/-^* monocytes, mixed chimeric mice were generated by reconstitution of CD45.2 wt mice with a 1:1 mixture of wt (CD45.1) and *Il17ra^-/-^* (CD45.2) bone marrow. We first investigated possible contamination with host (CD45.2) cells among circulating blood monocytes by assessing *Il17ra* expression (figure 2). It was virtually absent on circulating CD45.2 monocytes indicating very good replacement of recipient cells. *Il17ra* expression was significantly higher on wt Gr1^high^ than Gr1^low^ monocytes (figure 2B). *Il17ra* associates with *Il17rc* and both are required for IL-17A and IL-17F signaling [18], [19], but in combination with *Il17rb*, it also serves as a receptor of IL-25 (IL-17E) [26]. We found high expression of *Il17ra* mRNA, and lower levels of *Il17rb*, *Il17rc* and *Il17re* (required for IL-17C signal [27]) in bone marrow monocytes before Gr1^high^ to Gr1^low^ transition (figure 2C).

**Figure 3:**
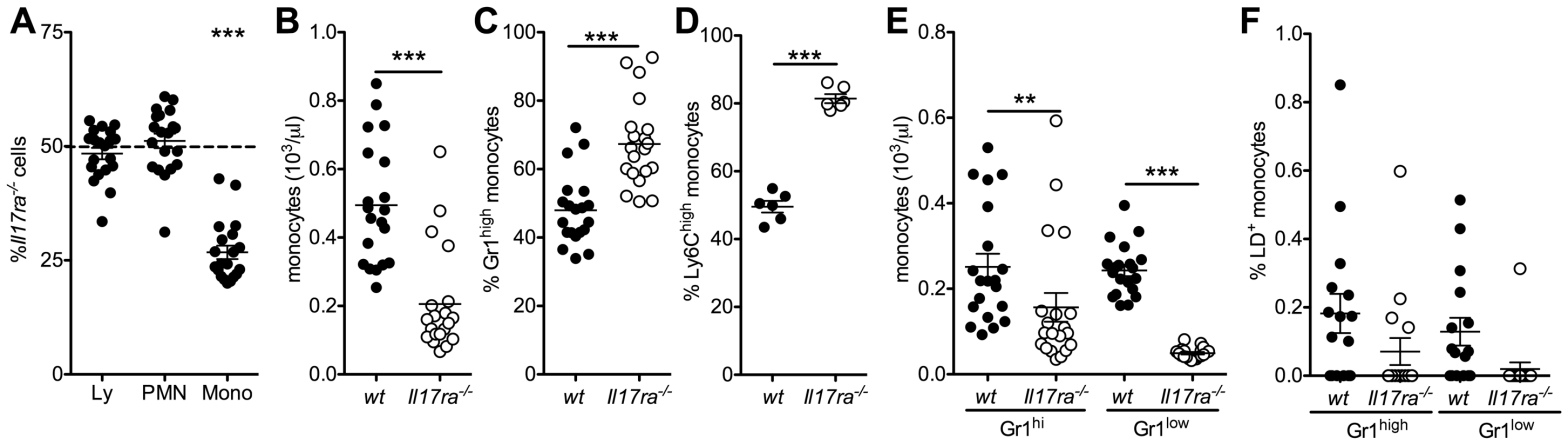
Absence of IL-17 receptor A reduces monocyte counts in a cell specific manner. Leukocyte analysis in mixed bone marrow chimeric wt (CD45.1)/*Il17ra^-/-^* (CD45.2) mice. (**A**) The proportion of *Il17ra^-/-^* cells among blood lymphocytes (Ly), granulocytes (PMN) and monocytes (mono). (**B**) Absolute monocyte concentrations for each genotype. (**C,D**) The proportion of Gr1^high^ (C, n = 20 mice from 3 independent transplantations) and Ly6C^high^ monocytes (D, n = 6) was significantly higher among *Il17ra^-/-^* than wt cells. (**E**) Absolute blood monocyte concentrations (Bonferroni after One-way-ANOVA, n = 20 mice from 3 independent transplantations). (**F**) Proportion of blood monocytes staining as dead cells (n = 16 from 3 independent transplantations, Bonferroni after One-way-ANOVA)).

We next assessed the proportion of *Il17ra^-/-^* cells among blood leukocytes (figure 3). In individual bone marrow chimeric wt/*Il17ra^-/-^* animals, wt and *Il17ra^-/-^* proportions were equal among lymphocytes and granulocytes, but significantly decreased among monocytes (figure 3A). Consistently, peripheral blood concentrations of *Il17ra^-/-^* monocytes were significantly lower than wt monocytes (figure 3B). The proportion of Gr1^high^ monocytes was significantly higher among *Il17ra^-/-^* than wt monocytes in each individual mouse (figure 3C). Principally the same results were found using the Ly6C antibody (figure 3D). This was reflected by a much more marked decrease in absolute *Il17ra^-/-^*Gr1^low^ monocyte than Gr1^high^ monocyte blood concentrations compared to wt cells in identical environments (figure 3E). There was no significant difference in the proportion of dead monocytes of either genotype or subtype in the circulation (figure 3F), only a non-significant tendency towards even less cell death in *Il17ra^-/-^*Gr1^low^ monocytes.

These data suggest a deficiency in monocytes, most markedly Gr1^low^ cells, in the absence of IL-17 signal. The fact that this was even more marked in an identical environment suggests a role of the IL-17 signal on a cellular level.

### IL-17ra^-/-^ monocyte derived macrophage generation under homeostatic conditions

**Figure 4:**
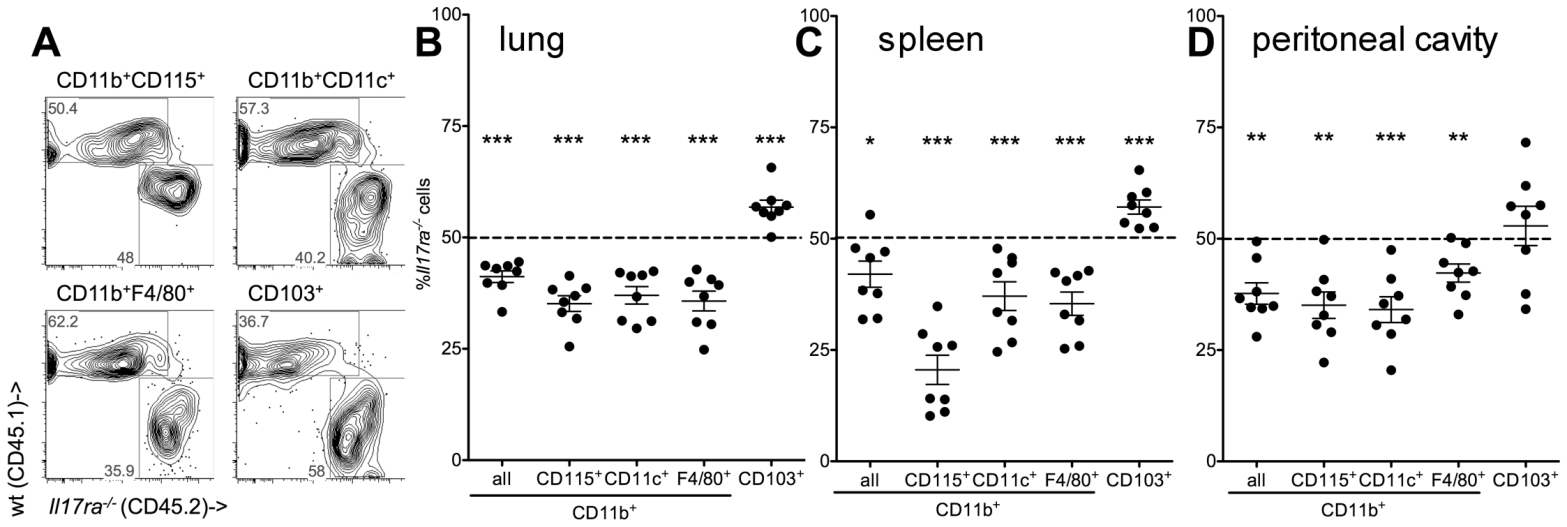
Homeostatic tissue macrophages are altered without IL-17 receptor. (**A–D**) Monocytes, macrophages and dendritic cells from mixed bone marrow chimeric wt/*Il17ra^-/-^* mice were analyzed by flow cytometry after enzymatic digestion of lungs (examples in A, statistical analysis in B), in spleen (C) and peritoneal cavity (D) (n = 8 and 2 independent transplantations, paired t-tests).

As there was no indication of enhanced cell death in *Il17ra^-/-^*Gr1^low^ monocytes, we next tested whether or not their deficiency in blood was due to increased migration into tissues. Gr1^low^ monocytes are known precursors of pulmonary tissue macrophages [15]. Flow cytometric analysis of lungs of mixed chimeric wt/*Il17ra^-/-^* mice revealed that *Il17ra^-/-^* cells were underrepresented in pulmonary CD115^+^ monocytes and both CD11b^+^F4/80^+^ and CD11b^+^CD11c^+^ macrophages (figure 4A,B). This does not suggest an increased migration of the gene deficient cells into the tissue. The results for the CD11b^+^ myeloid compartment were significantly different from the findings in CD103^+^CD11b^-^ non-myeloid dendritic cells. Here, no decrease in CD45.2 cells was noted (figure 4A,B). Indeed, there was a relative increase over CD45.1, which might be an *Il17ra^-/-^* phenotype but would also be expected in any cell type not completely replaced after lethal irradiation due to residual recipient cells.

Macrophages were also assessed in the spleen and resting peritoneal cavity. Also in these compartments, the proportion of *Il17ra^-/-^* among all CD11b^+^ cells and among macrophages in mixed bone marrow chimeric wt/*Il17ra^-/-^* mice was significantly lower than the expected 50% (figure 4C,D).

These data suggest the IL-17 signal at the level of the individual cell to be involved in tissue myeloid macrophage homeostasis in the absence of inflammation.

### Bone marrow Gr1^low^ monocyte formation

**Figure 5:**
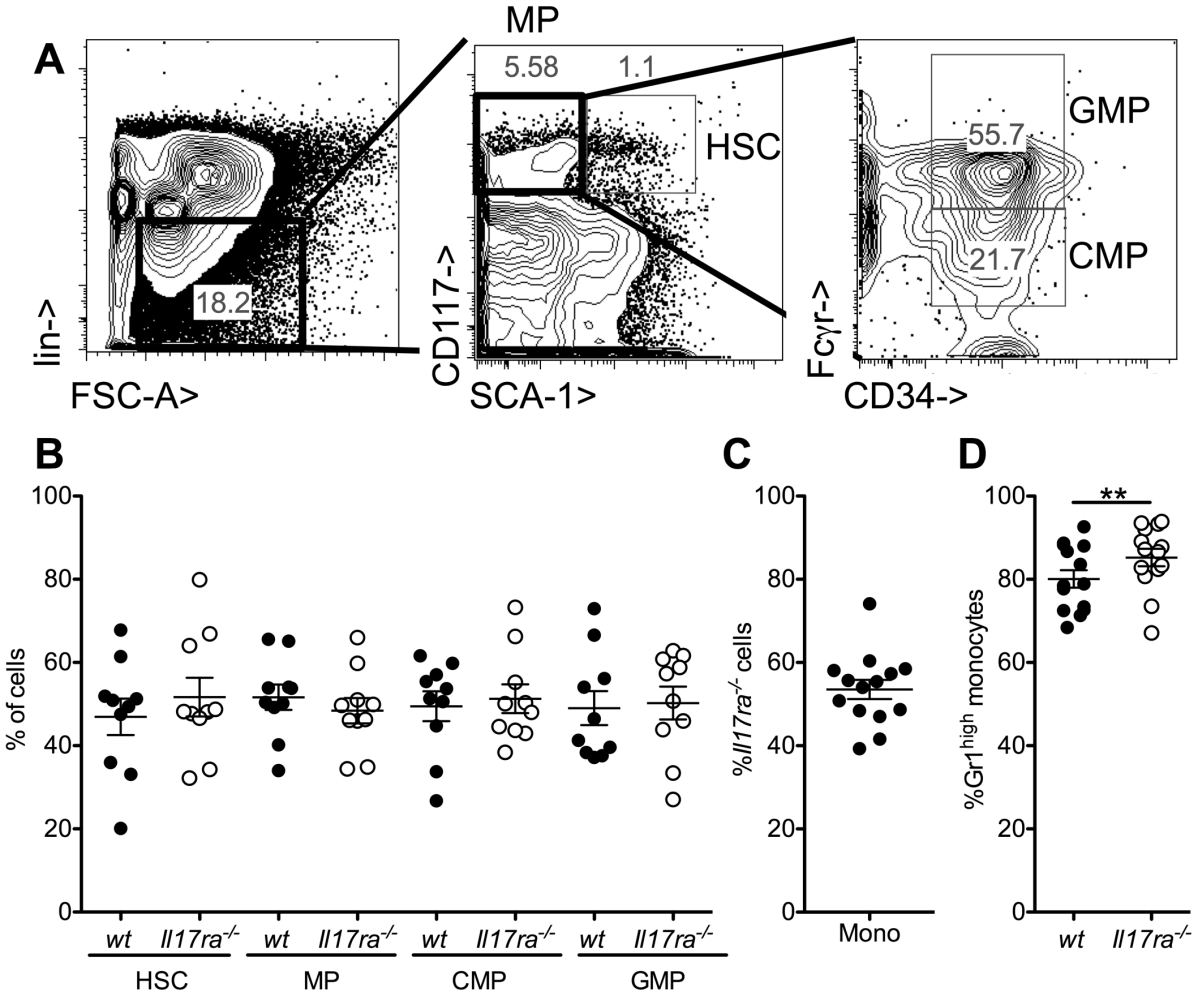
*Il17ra^-/-^*Gr1^low^ monocyte development in the bone marrow. (**A,B**) Bone marrow progenitors were defined among lineage negative cells as hematopoietic stem cells (HSC, CD117^+^Sca^+^), myeloid progenitors (MP, CD117^+^Sca^−^), common myeloid progenitors (CMP, CD34^+^Fcγr^−^ among MP) and granulocyte macrophage progenitors (GMP, CD34^+^Fcγr^+^ among MP). To assess the role of the IL-17 receptor on a cell specific level, the proportion of wt and *Il17ra^-/-^* cells at each stage was analyzed in mixed bone marrow chimeric mice (B, n = 10 from 3 independent transplantations). (**C,D**) Similarly bone marrow CD11b^+^CD115^+^ monocytes were analyzed for genotype (C) and Gr1^high^ proportion (D) in wt and *Il17ra^-/-^* cells (n = 10 from 3 independent transplantations, paired t-test).

We next tested whether the observed monocyte defect was due to a defect in monocyte generation in the bone marrow. To this end, bone marrow myeloid cell maturation and monocytes were further investigated in wt/*Il17ra^-/-^* mixed chimeras to provide identical environments for development (figure 5). Bone marrow progenitors, defined as described [28], were very similar for the genotypes (figure 5A,B). Similarly, the proportion of *Il17ra^-/-^* CD11b^+^CD115^+^ monocytes in the bone marrow did not differ from the expected 50% (figure 5C). However a small, but very consistent and significant increase in the proportion of *Il17ra^-/-^*Gr1^high^ cells was observed (figure 5D).

These data show that the decrease in the proportion of Gr1^low^ *Il17ra^-/-^* monocytes commences at the bone marrow level.

### Peripheral Gr1^low^ monocyte recovery

**Figure 6:**
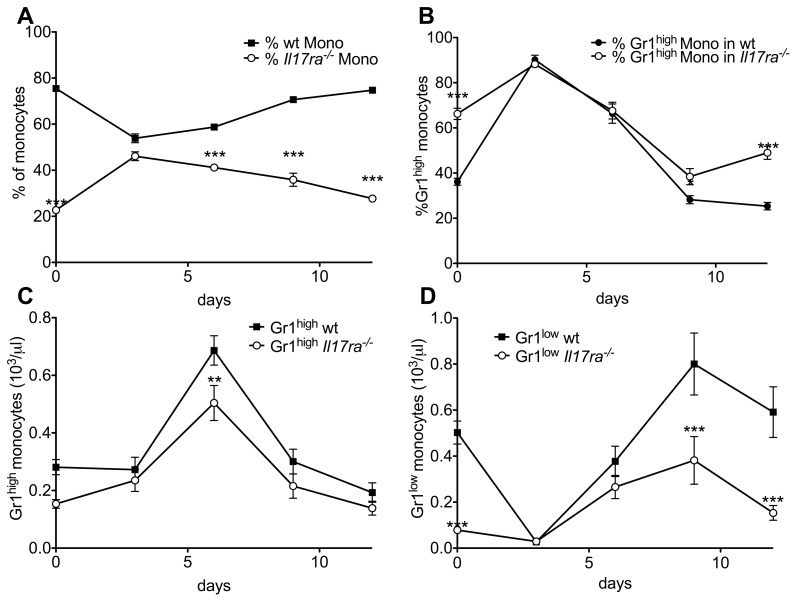
Lack maintenance of *Il17ra^-/-^*Gr1^low^ monocyte population after normal initial recovery. (**A-D**) Mixed bone marrow chimeric wt/*Il17ra^-/-^* mice underwent monocyte depletion by liposomal clodronate. During recovery, monocyte genotype (A), proportion of Gr1^high^ and Gr1^low^ cells among the monocytes of each genotype (B) and absolute blood concentrations of Gr1^high^ (C) and Gr1^low^ monocytes (D) of each genotype were measured (n = 8, 2 independent transplantations, Bonferroni after two-way-ANOVA).

Given the unchanged total monocyte population in the bone marrow, we investigated monocyte repopulation after depletion with liposomal clodronate in mixed chimeric wt/*Il17ra^-/-^* mice [24]. As described, clodronate completely abolished peripheral blood monocyte counts on day one after application (data not shown). During initial recovery on day three, the proportion of *Il17ra^-/-^* monocytes was equal to wt cells (figure 6A), as was the proportion of Gr1^high^ cells in both genotypes (figure 6B) which is reflected in similar absolute counts of the monocyte subtypes of either genotype at this timepoint (figures 6C,D). This suggests no major deficiency in initial monocyte recovery. However, during the following days, which brought normalization of wt absolute monocyte counts of each subgroup and the proportion of Gr1^high^ cells ([10], figure 6), *Il17ra^-/-^* cell counts decreased, largely due to a stop in the rise of absolute numbers of Gr1^low^ cells (figure 6D).

These data suggest no defect in initial generation of Gr1^low^ monocytes, but rather in maintenance of their numbers as the reason for low *Il17ra^-/-^* monocyte counts.

### Monocyte Gr1^high^ to Gr1^low^ subpopulation transition in the absence of IL-17 receptor A

**Figure 7:**
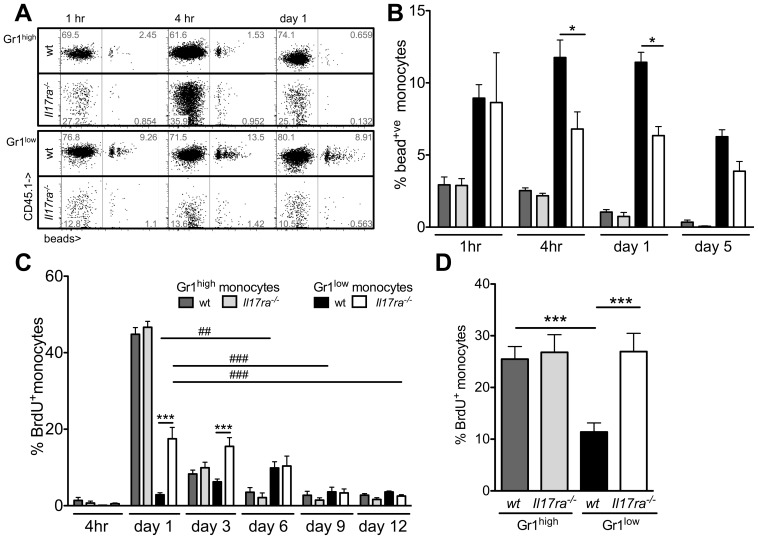
Kinetics of labeled Gr1^high^ and Gr1^low^ monocytes in the presence and absence of *Il17ra^-/-^*. (**A,B**) Monocytes in mixed chimeric wt/*Il17ra^-/-^* mice were labeled with fluorescent latex beads (A). The proportion of labeled CD11b^+^CD115^+^ cells among each monocyte subgroup (Gr1^high^ and Gr1^low^ monocytes) within each genotype was assessed over time (B, n = 4-5, 2 independent transplantations, Bonferroni after ANOVA). (**C**) Dividing cells in mixed bone marrow chimeric wt/*Il17ra^-/-^* mice labeled with a single BrdU injection and peripheral blood monocytes were analyzed for BrdU incorporation at the indicated timepoints. In every animal, the proportion of labeled cells among Gr1^high^ and Gr1^low^ monocytes of either genotype was assessed over time (n = 4, * indicates sign. differences between wt and *Il17ra^-/-^* Gr1^low^ monocytes at the indicated timepoints, # indicates a sign. change over time within wt (black bars) or *Il17ra^-/-^* (white bars) Gr1^low^ monocytes, analysis with Bonferroni after ANOVA). (**D**) Proliferation of bone marrow Gr1^high^ and Gr1^low^ monocytes of both genotypes was assessed 20 h after BrdU injection (n = 7 from 2 independent transplantations, Bonferroni after ANOVA).

Previous reports have suggested that Gr1^high^ to Gr1^low^ monocyte conversion occurs in the periphery [10], [11]. To investigate Gr1^high^ to Gr1^low^ monocyte conversion in the absence of IL-17 receptor A, we used fluorescent bead labeling [13] in mixed chimeric mice. Initial labeling of both subsets and bead clearance from Gr1^high^ cells was similar for both genotypes (figure 7A,B). Consistent with conversion of Gr1^high^ to Gr1^low^ monocytes and as described, a delayed rise in the proportion of bead^+^Gr1^low^ cells occurred in wt cells (figure 7B) [13]. However, this was completely absent in Gr1^low^ *Il17ra^-/-^* monocytes in an identical environment.

To assess monocyte subtype conversion with an independent stable cell labeling method, proliferating cells in resting mixed chimeric mice were pulse labeled with BrdU and peripheral monocyte BrdU content assessed over time (figure 7C). The proportion of BrdU^+^ among Gr1^high^ monocytes of both genotypes rose simultaneously during the first day and decreased afterwards. This is consistent with the similar proliferation of bone marrow monocytes (figure 7D) and suggests no difference in liberation from the bone marrow. Among Gr1^low^ monocytes of wt genotype, the rise in the BrdU^+^ proportion was sustained until day 6, likely due to repopulation by peripheral conversion of Gr1^high^ cells. In contrast, among the low absolute numbers of Gr1^low^ *Il17ra^-/-^* monocytes, only an initial rise in the BrdU^+^ proportion on day one was observed, similar to Gr1^high^ monocytes of either genotype. No delayed rise as in the Gr1^low^wt monocyte population occurred, the proportion of pulse-labeled Gr1^low^ *Il17ra^-/-^* monocytes rather steadily declined. Also in the bone marrow, among the reduced number of Gr1^low^ *Il17ra^-/-^* monocytes (figure 5D), the proportion of BrdU^+^ cells was significantly higher than of wtGr1^low^ monocytes and similar to what was observed in Gr1^high^ monocytes of either genotype (figure 7C). This can account for the higher initial proportion of blood BrdU^+^ among Gr1^low^ *Il17ra^-/-^* than wt monocytes and indicates a larger proportion of recent cell divisions in this group.

These data suggest that Gr1^high^ to Gr1^low^ monocyte conversion is altered in the absence of *Il17ra*.

### IL-17ra^-/-^ macrophage generation in peritonitis

**Figure 8:**
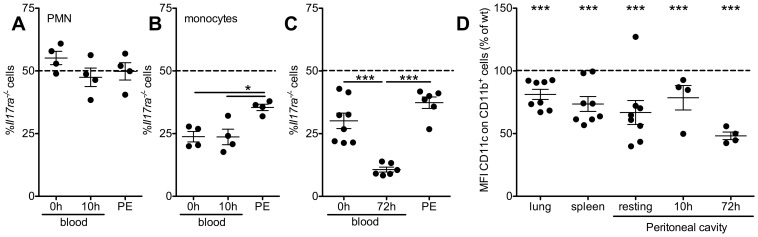
In peritonitis, *Il17ra^-/-^* monocyte recruitment is sustained, but macrophage phenotype changed. (**A,B**) Peritonitis was induced in mixed bone marrow chimeric wt/*Il17ra^-/-^* mice. Peripheral blood (at start and end of the experiment) and peritoneal neutrophils (PMN, CD11b^+^CD115^−^Gr1^high^, A) and monocytes (CD11b^+^CD115^+^, B) were analyzed by flow cytometry after 10 h (n = 4, Bonferroni after ANOVA). (**C**) At day 3, intraperitoneal myeloid CD11b^+^ cells were analyzed and compared to peripheral blood monocytes at start and end of the experiment (n = 4–6, 2 independent transplantations, Bonferroni after ANOVA). (**D**) CD11c expression on wt and *Il17ra^-/-^* myeloid (CD11b^+^) cells was compared in lung, spleen, resting and inflamed peritoneal cavity of mixed bone marrow chimeric wt/*Il17ra^-/-^* mice (n = 4–8, paired t-tests).

Monocyte subtypes are differentially recruited to sites of inflammation. We studied peritonitis as a model of preferential Gr1^high^ monocyte accumulation and acute inflammatory macrophage accumulation. Consistently, after 10 h of inflammation, neither the proportion of *Il17ra^-/-^* PMN nor of *Il17ra^-/-^* monocytes in the peritoneal cavity was lower than in peripheral blood at the start of the experiment (figure 8A,B). *Il17ra^-/-^* monocytes were even overrepresented in the peritoneal infiltrate (figure 8B). After three days of inflammation, the proportion of wt and *Il17ra^-/-^*CD11b^+^ macrophages in the peritoneum was not different from peripheral blood before induction of inflammation, while blood *Il17ra^-/-^* monocytes were depleted (figure 8C). While Gr1^high^ and Gr1^low^ monocytes were no longer distinguishable in peritoneal inflammation, we further analyzed CD11c that is expressed on Gr1^low^ monocytes and on some macrophage subtypes such as pulmonary and vascular macrophages [1]–[3], [29]. Compared to wt cells from the same mouse, CD11c expression was significantly lower on *Il17ra^-/-^* cells (figure 8D), similarly to what was previously observed in complete *Il17ra^-/-^* mice [21].

### Role of myeloid IL-17ra^-/-^ in renal macrophage accumulation and fibrosis

**Figure 9:**
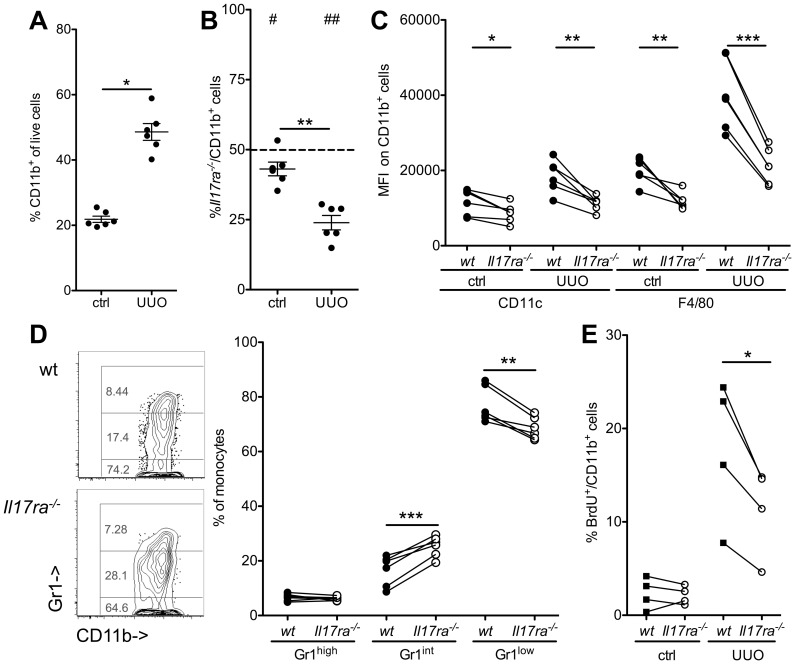
Reduced renal macrophage generation in response to urethral obstruction in the absence of IL-17 receptor. Renal fibrosis was induced by unilateral ureteral obstruction (UUO) in mixed wt/*Il17ra^-/-^* bone marrow chimeric mice. Myeloid cell accumulation in the obstructed kidney was assessed for wt and *Il17ra^-/-^* cells in identical environments (**A,B**) Total CD11b^+^ myeloid cell infiltration to the kidney (A, ctrl = contralateral kidney, UUO = obstructed kidney) and their genotype (B, #indicates significance level against expected 50% wt ratio, *between UUO and ctrl) was determined. (**C,D**) For assessment of differentiation, renal myeloid cell CD11c, F4/80 (C) and Gr1 (D) surface marker expression was tested (n = 6 from 2 independent transplantations, paired t-tests). (**E**) Renal myeloid cell proliferation was assessed 24 h after a single injection of BrdU (n = 4).

To test whether the decrease in Gr1^low^ monocytes was functionally relevant, we investigated kidney injury induced by unilateral ureteral obstruction (UUO) that induces macrophage accumulation and renal fibrosis [30], [31]. To assess the IL-17 effect on individual myeloid cells, renal cell infiltration was investigated in mixed chimeric wt/*Il17ra^-/-^* mice seven days after obstruction surgery (figure 9). UUO significantly enhanced renal CD11b^+^ leukocyte accumulation (figure 9A). Similar to other organs (figure 4), the proportion of CD11b^+^ *Il17ra^-/-^* cells was already lower than wt in the unobstructed contralateral kidney. This was significantly enhanced in obstructive inflammation (figure 9B). Differential recruitment was mainly due to monocytes and not neutrophils, which were 39±15% *Il17ra^-/-^*(p = 0.13, n = 6, 2 independent transplantations). Obstruction also increased CD11c and F4/80 expression on myeloid cells at day 7 (figure 9C). This phenotype was significantly less pronounced in the cells deficient in IL-17 signaling. A pro-fibrotic profile has been described for Gr1^high^-derived Gr1^low^ monocytes/macrophages in renal fibrosis [32]. Among *Il17ra^-/-^* cells, the proportion of these Gr1^low^ cells was significantly lower than in wt cells in the same environment (figure 9D). UUO markedly increased proliferation of renal leukocytes (figure 9E). This was significantly dampened in the absence of *Il17ra*.

**Figure 10:**
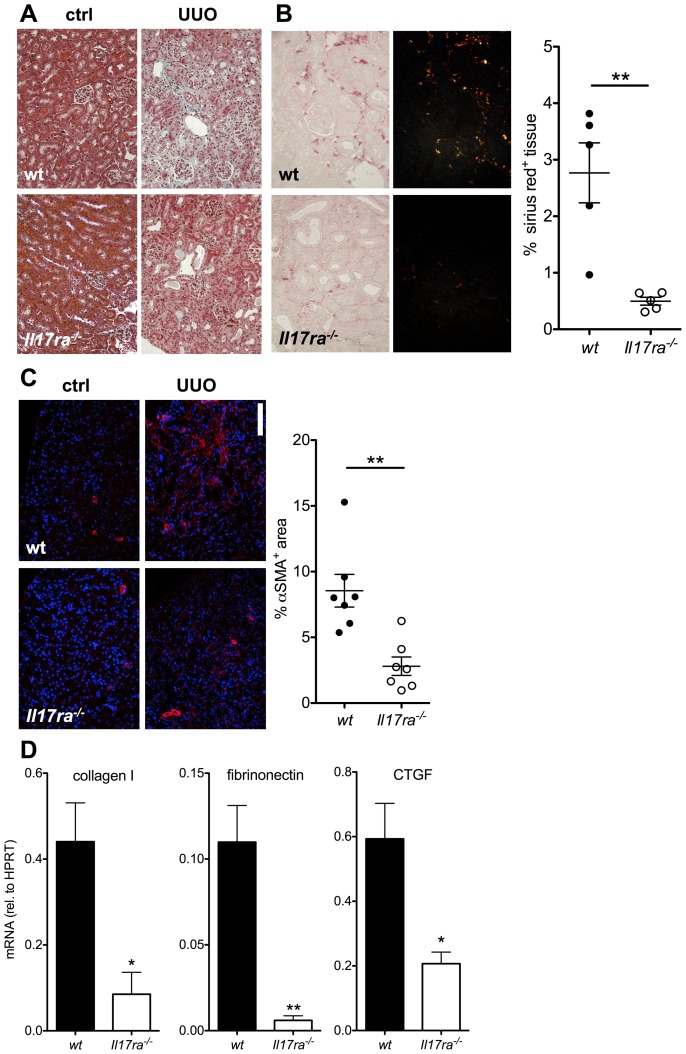
Decreased renal fibrosis in the absence of IL-17 receptor on myeloid cells. Unilateral ureteral obstruction was performed in wt and *Il17ra^-/-^* bone marrow chimeric mice. (**A–C**) Histologic assessment of fibrosis was conducted after Masson's trichrome (A), Sirius red (B, birefringent sirius red positive fibrotic tissue area analyzed from n = 5/group from 2 independent transplantations, 40× original magnification) and αSMA- immunofluorescent staining (C, n = 7 from 2 independent transplantations, scale bar = 100 µm) on day 7. (**D**) mRNA expression of collagen I, fibrinonectin and CTGF was assessed in obstructed kidneys by quantitative PCR (day seven, n = 3, t-tests).

Renal histology was assessed in mice reconstituted with either wt or *Il17ra^-/-^* bone marrow (figure 10). The proportion of fibrotic tissue area assessed with Sirius red staining and anti-αSMA staining was significantly lower in the absence of bone marrow *Il17ra* (figure 10A–C). mRNA expression of the pro-fibrotic molecules collagen I, fibronectin and CTGF was also significantly decreased (figure 10D). Leukocyte infiltration is difficult to compare in kidney at different stages of fibrosis, however, there was a tendency towards less absolute CD45^+^ and CD11b^+^ leukocyte infiltration in the absence of myeloid *Il17ra* in complete bone marrow chimeric mice (data not shown). These data suggest that lack of IL-17 receptor A on the myeloid cell level significantly alters inflammation and diminishes fibrosis in renal obstruction.

## Discussion

Our data demonstrate a role for IL-17 receptor in maintenance of the Gr1^low^ monocyte population and macrophages *in vivo*.

Most markedly in mixed chimeric mice, where cell-specific effects can be tested, *Il17ra^-/-^*Gr1^low^ monocyte numbers were decreased. Different from the monocyte phenotype observed in M-CSF receptor CD115 blockade or M-CSF scavenging [7], [8] or deficiency in nuclear receptor NUR77 [9] we did not observe significant differences in monocyte cell death between the genotypes. Also, bone marrow progenitors and initial recovery after monocyte depletion was unaltered.

Our data might suggest a different mechanism how IL-17 modulates monocyte counts; Gr1^high^ to Gr1^low^ monocyte passage was altered and appeared defective in *Il17ra^-/-^* monocytes. While a specific genetic marker that could indicate the passage from Gr1^high^ to Gr1^low^ is not currently available, we made efforts to study this phenomenon as directly as possible with two independent methods, BrdU pulse-labeling and injection of latex beads [13] with very similar results. Consistent with this interpretation of the data, bone marrow *Il17ra^-/-^*Gr1^low^ monocytes had more recently proliferated than wtGr1^low^ cells which would be expected in the absence of or at strongly reduced rates of cells converted from Gr1^high^ cells. Interestingly, initial *Il17ra^-/-^*Gr1^low^ monocyte recovery after monocyte depletion was normal. This is consistent with the hypothesis that some Gr1^low^ monocytes may be different from the large proportion of Gr1^low^ monocytes that is observed in wt cells under resting conditions regarding their requirement of the *Il17ra* signal. Genetic labeling techniques will be required to further test this proposition.

Gr1^low^ monocytes are precursors of pulmonary resident macrophages [12], [15]. Indeed, we found that the proportion of *Il17ra^-/-^* cells was decreased among resident pulmonary and also splenic and peritoneal macrophages. Some resident macrophages may be slow-replicating and even not radiosensitive [12], however with our transplantation strategy, residual wt cells would by grouped together with the *Il17ra^-/-^* cells and thereby obscure and not increase a *Il17ra^-/-^* specific phenotype. In many forms of acute monocytic inflammation, mostly Gr1^high^ monocytes are recruited [5]. Consistently, in our experiments in peritonitis in mixed chimeric wt/*Il17ra^-/-^* mice, peritoneal CD115^+^ cell numbers were equal for both genotypes. This difference from complete *Il17ra^-/-^* and *Il17a^-/-^* mice, where total cell numbers were reduced [21], may be due to lack of IL-17 induced second messengers such as chemokines from e.g. epithelial cells [18]. In contrast, CD11c, which is expressed on Gr1^low^ monocytes [1]–[3], was decreased on peritoneal *Il17ra^-/-^*CD11b^+^ myeloid cells in both, fully gene deficient mice and mixed chimeras. Similar results were found in UUO. This suggests that myeloid CD11c expression modulation by IL-17 is cell intrinsic.

*Il17ra* is required for at least IL-17A, IL-17F, IL-17C and IL-17E signal [19]. Further work on mice deficient in one or more the *Il17ra* binding cytokines is required to elucidate whether all of them are required or they can substitute for another at baseline and/or specific inflammatory conditions.

Monocytes and monocyte-derived macrophages are also important for host response to infections [1]–[3]. Peritoneal listeriosis is an infection that induces Gr1^low^ before Gr1^high^ monocyte infiltration [17]. Our finding that *Il17ra* promotes Gr1^low^ monocyte counts may provide a mechanism for the fact that IL-17 mediates immunity against this pathogen and IL-17 enhances antigen presenting cell function [33]. Our data showing that *Il17ra* enhances myeloid CD11c expression, a marker of dendritic cells in mice, is consistent with a direct IL-17 effect.

Not only beneficial inflammation, but also tissue destruction in fibrosis is associated with macrophage accumulation. We investigated the effect of *Il17ra* on myeloid cells in renal fibrosis and found a decrease in *Il17ra^-/-^* macrophages in direct comparison of wt and *Il17ra^-/-^* cells in mixed chimeric mice. This is reminiscent of anti-M-CSF receptor therapy that inhibits macrophage accumulation in renal inflammation in ureteral obstruction [34], lupus nephritis, [35] and diabetic nephropathy [36]. In addition to reduced numbers, their phenotype was altered. In UUO, Gr1^high^-derived Gr1^low^ monocytes/macrophages have been shown to express pro-fibrotic mediators [32]. Our results show that this group was significantly decreased in the absence of *Il17ra*. Our study prompts further investigation of functional differences between wt and *Il17ra^-/-^* monocytes and macrophages. This will also have to take into account possible compensatory mechanisms in the complete absence of *Il17ra*. Blood monocyte TNFα production in response to LPS was very similar in wt and *Il17ra^-/-^* in the same environment (data not shown) and phagocytosis of latex beads was very similar (figure 7), however, this may be altered for different substrates and during macrophage differentiation.

Recent reports propose IL-17A as an important mediator in non-renal fibrosis, bleomycin induced pulmonary fibrosis [37], [38] and hepatic fibrosis induced by either cholestasis or hepatotoxic agents [39]. Interestingly, the decrease in hepatic fibrosis was completely mediated by *Il17ra* deficiency on hematopoietic cells [39]. Also in the kidney, our experiments show a highly significant decrease in fibrosis in mice deficient only in bone-marrow *Il17ra*. Our finding that *Il17ra* modulates myeloid cell infiltration and phenotype in renal fibrosis may therefore represent a mechanism also for the hepatic disease. As IL-17 receptor ligands are soluble serum factors, blockade should be investigated as a therapeutic targets in the treatment of pro-inflammatory disease processes in tissue fibrosis.

In summary, our data demonstrate *Il17ra* a novel regulator of monocyte homeostasis and macrophage development during fibrosis *in vivo*.
